# Identification of Immune Microenvironment Changes and the Expression of Immune-Related Genes in Liver Cirrhosis

**DOI:** 10.3389/fimmu.2022.918445

**Published:** 2022-07-12

**Authors:** Yuwei Liu, Yutong Dong, Xiaojing Wu, Xiaomei Wang, Junqi Niu

**Affiliations:** ^1^ Department of Hepatology, Center of Infectious Diseases and Pathogen Biology, The First Hospital of Jilin University, Changchun, China; ^2^ Key Laboratory of Zoonosis Research, Ministry of Education, The First Hospital of Jilin University, Changchun, China; ^3^ Key Laboratory of Organ Transplantation, Ministry of Education, The First Hospital of Jilin University, Changchun, China

**Keywords:** cirrhosis, single-cell RNA sequencing, immunity-related genes, differentially expressed genes, immune microenvironment

## Abstract

Liver inflammation and the immune response have been recognized as critical contributors to cirrhosis pathogenesis. Immunity-related genes (IRGs) play an essential role in immune cell infiltration and immune reactions; however, the changes in the immune microenvironment and the expression of IRGs involved in cirrhosis remain unclear. CD45+ liver cell single-cell RNA (scRNA) sequencing data (GSE136103) from patients with cirrhosis were analyzed. The clusters were identified as known cell types through marker genes according to previous studies. GO and KEGG analyses among differentially expressed genes (DEGs) were performed. DEGs were screened to identify IRGs based on the ImmPort database. The protein-protein interaction (PPI) network of IRGs was generated using the STRING database. IRGs activity was calculated using the AUCell package. RNA microarray expression data (GSE45050) of cirrhosis were analyzed to confirm common IRGs and IRGs activity. Relevant regulatory transcription factors (TFs) were identified from the Human TFDB database. A total of ten clusters were obtained. CD8+ T cells and NK cells were significantly decreased in patients with cirrhosis, while CD4+ T memory cells were increased. Enrichment analyses showed that the DEGs focused on the regulation of immune cell activation and differentiation, NK-cell mediated cytotoxicity, and antigen processing and presentation. Four common TFs, IRF8, NR4A2, IKZF3, and REL were expressed in both the NK cluster and the DEGs of liver tissues. In conclusion, we proposed that the reduction of the CD8+ T cell cluster and NK cells, as well as the infiltration of CD4+ memory T cells, contributed to immune microenvironment changes in cirrhosis. IRF8, NR4A2, IKZF3, and REL may be involved in the transcriptional regulation of NK cells in liver fibrosis. The identified DEGs, IRGs, and pathways may serve critical roles in the development and progression of liver fibrosis.

## Introduction

Liver cirrhosis is the irreversible form of liver fibrosis. It was the 11th highest contributor to global mortality from 2000 to 2019 according to the WHO (1). Liver fibrosis is a common and complex pathological pathway that results from diverse liver injuries. Pathological, persistent liver injury leads to hepatocyte necrosis and hepatic stellate cell (HSC) activation which can result in distortion of hepatic architecture, nodular formation, and excessive extracellular matrix (ECM) production. When hepatic architecture is dysregulated and excessive nodules occur, liver fibrosis converts to cirrhosis with progressive loss of liver function. In recent years, liver inflammation and liver immune microenvironment changes have been recognized as critical contributors to cirrhosis pathogenesis (2, 3). Accumulating experimental evidence has revealed that the immune cells can regulate both the progression and regression of liver fibrosis.

During the fibrogenic process, the immune system participates in wound healing and tissue repair by initiating inflammation. After liver injuries, the infiltrated immune cells are recruited to the site of injured hepatocytes and contribute to the liver fibrotic cascade by secreting pro-inflammatory cytokines such as TNF-α, IL-6, and CCL4 (4). These cytokines mediate the crosstalk between immune cells and HSCs, which leads to HSC activation and transdifferentiation to myofibroblasts. Some cytokines such as IFN-γ, can also regulate ECM synthesis and remodelling. For viral hepatitis related fibrosis, the CD4+ T cell activity and CD8+ T cell cytotoxic effects to achieve viral clearance can directly mediate HSC activation and fibrogenesis (5). In addition, natural killer (NK) cells display anti-fibrotic activity by directly killing activated HSCs, inducing HSC apoptosis and cell cycle arrest (6). Immunity-related genes (IRGs) play essential roles in immune infiltration; however, the expression characteristics of IRGs and immune microenvironment changes in cirrhosis remain unclear.

Single-cell RNA (scRNA) sequencing technology advances have made it possible to isolate and determine the transcriptomic profiles of liver immune cells. This study investigated the expression characteristics of IRGs and immune microenvironment changes in cirrhosis by combining single-cell RNA (scRNA) and RNA microarray expression data.

## Methods

### ScRNA Sequencing Data Analysis

Published scRNA-seq data were retrieved from the Gene Expression Omnibus (GEO) dataset GSE136103 (7). Single-cell transcriptomic data of CD45+ liver leukocytes were chosen from the liver tissue of 5 healthy controls and 5 cirrhotic patients. The Seurat R package (Version 4.1.0) was used for downstream principal component analysis (PCA) and t-distributed stochastic neighbour embedding (t-SNE) analysis. Cells with <200 genes, >2,500 genes, or >5% mitochondrial genes were filtered out. A total of 30,934 filtered liver cells were selected for analysis. Gene expression was normalized using the “LogNormalize” method and further scaled. After data normalization, 2000 highly variable genes (HVGs) were identified using the Seurat “FindVariableGene” function with default parameters. Subsequently, PCA was applied to identify significant principal components (PCs), and the P value distribution was visualized using the “JackStraw” and “ScoreJackStraw” functions. Ultimately, fifteen PCs were selected for t-SNE analysis. The “FindClusters” function was used to classify the cells into twenty different clusters with a resolution of 0.5. The Seurat “FindAllMarkers” function with default parameters (logfc threshold = 0.5) was applied to identify marker genes for each cluster. Cell type identification was performed based on the marker genes in each cluster and manually checked according to previous studies (8, 9). The Seurat “FindMarkers” function with default parameters (logfc threshold = 0.25) was applied to identify differentially expressed genes (DEGs) between the healthy group and the cirrhotic group. The EnhancedVolcano R package (1.12.0) was used to visualize the DEGs between the two groups.

### RNA Microarray Expression Data Analysis

Raw data of GSE45050 were downloaded from the GEO database using the GEOquery R package (Version 2.62.2) (10). DEGs were calculated using the limma R package (Version 3.50.1). Genes with an adjusted P value <0.05, and an absolute logFC > 0.8 were considered DEGs. Volcano and heatmap plots were generated using the ggplot2 R package (Version 3.3.5).

### IRG Scoring

DEGs of scRNA data and RNA microarray expression data were screened separately to identify IRGs based on the ImmPort database (https://www.immport.org/shared/home), and IRGs were selected for IRG scoring with the AUCell R package (Version 1.16.0). According to the area under the curve (AUC) value of the selected IRGs, gene expression rankings of each cell were generated to estimate the highly expressed gene set proportion in each cell. Cells expressing more genes within the gene set had higher AUC values. The “AUCell_exploreThresholds” function was used to determine the threshold to identify gene set active cells. Then, the AUC score of each cell was mapped to the UMAP embedding using the ggplot2 R package to visualize the active clusters.

### GO and KEGG Enrichment Analysis

The DEGs in GSE136103 were analyzed by Gene Ontology (GO) and Kyoto Encyclopedia of Genes and Genomes (KEGG) enrichment analyses. The ClusterProfiler R package (Version 4.2.2) was used to visualize the GO and the KEGG pathway data.

### PPI Network Construction

Protein-protein interaction (PPI) network analysis was performed using STRING (https://string-db.org/). A functional network was constructed through Cytoscape (Version 3.9). The Cytoscape plug-in cytoHubba was used to select the hub genes based on the degree method.

## Results

### ScRNA Profiling of Liver Leukocytes in Cirrhosis

**Figure 1:**
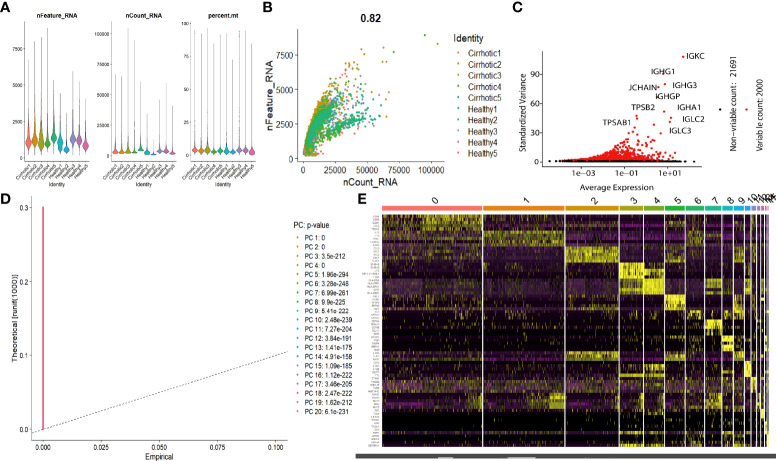
scRNA analysis of liver cirrhosis. **(A)** The gene features, gene counts, and mitochondrial gene percentage of each sample. **(B)** Correlation between genes and counts in each sample. **(C)** HVGs are colored red, and the top 10 HVGs are labeled. **(D)** PCs selection using the JackStraw function. **(E)** Heatmap of the top 5 DEGs in each cluster. The top 5 DEGs are labeled in yellow.

**Figure 2:**
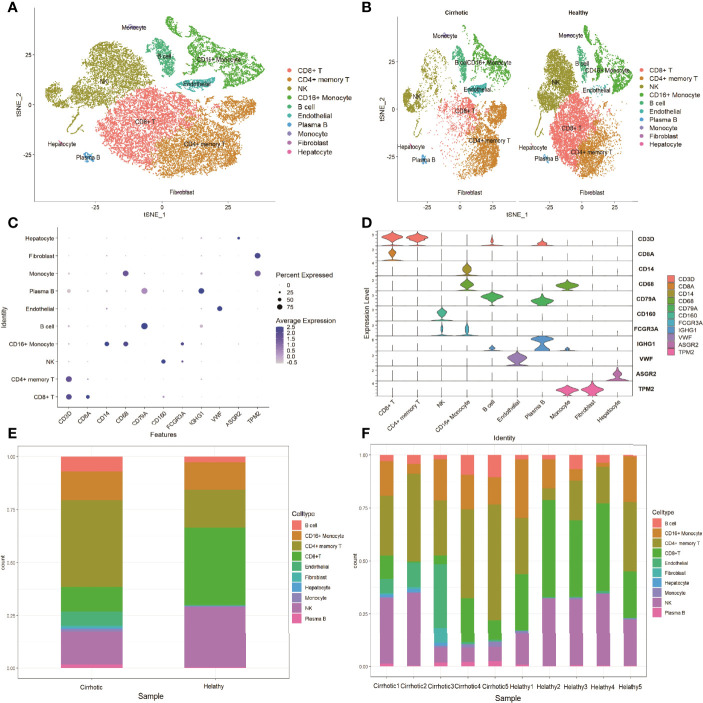
Marker gene expression of each cluster. **(A)** tSNE projection of all liver CD45+ leukocytes. Different cell types were colored with unique colors. **(B)** tSNE projection of the cirrhotic group and the control group. **(C)** Dot plot of cell type marker genes. Cell specific marker genes were selected according to previous studies. The color of the dots represents the average expression, and size of dots represents average percentage of cells expressing the selected gene. **(D)** Violin plot depicts the distributions of cell type marker genes in each cluster using density curves. The width of each violin plot corresponds to the frequency of cells with relevant gene expression levels. **(E)** Cluster distribution in the two groups. **(F)** Cluster distribution in each sample.

The scRNA sequencing dataset (GSE136103) from the GEO database was analyzed, which included CD45+ liver leukocytes, comprising 15,462 cells from liver cirrhosis patients and 21,779 cells from controls. After filtration, 30,934 cells comprising 11,974 cells from cirrhotic patients and 18,960 cells from healthy controls were retained. The expression characteristics of each sample are shown in (**Figure 1A**). nCount_RNA, which represents the number of unique molecular identifiers (UMI), positively correlated with nFeature_RNA, which represents the number of genes, with a correlation coefficient of 0.82 (**Figure 1B**). The top 10 hypervariable genes (HVGs) were identified (**Figure 1C**). IGKC and IGHG1 are the top two HVGs, which encode allotypes of immunoglobulin that regulate antigen-binding activity and immunoglobulin receptor binding activity (11). PCA identified all 20 PCs with the *P value <*0.05, as visualized with JackStrawPlot (**Figure 1D**). Sixteen separate clusters were identified using 10 PCs, and the top 5 marker genes of each cluster are listed (**Figure 1E**). These clusters could be identified as known cell lineages through marker genes, according to a previous study (8, 9). The ten clusters were visualized using the t-SNE algorithm (**Figure 2A**). Compared with the healthy group, the CD8+ T cluster and NK cluster had a significantly lower frequency of their cells in the cirrhotic group. The CD4+ memory T cluster had an increased percentage of CD4+ memory T cells in the cirrhotic group. (**Figure 2B**). The expression of cell type marker genes is shown in the dot plot (**Figure 2C**) and violin plot (**Figure 2D**). The cell proportions of each cluster in two groups are shown in **Figure 2E**. The number and proportion of each cluster in each sample are shown in **Figure 2F** respectively. The CD8+ T cluster and NK cluster were significantly reduced in the cirrhotic group compared with the healthy group (11.6% vs. 36.4%, 15.7% vs. 28.7%), while the CD4+ T cells percentage was increased in the cirrhotic group (41.0% vs. 17.8%).

### DEGs of Liver Cirrhosis and Enrichment Analysis

**Figure 3:**
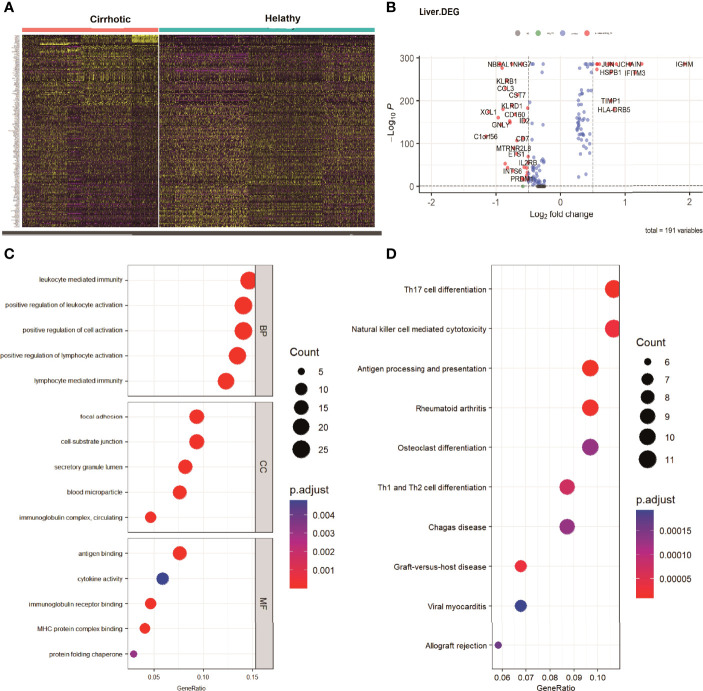
DEGs of cirrhosis from scRNA sequencing data. **(A)** Heatmap of all the DEGs. **(B)** Volcano plot (|logFC| > 0.25 and adjusted P value < 0.05).The DEGs are colored red. **(C)** GO analysis of DEGs. The top 5 biological processes (BP), the top 5 cellular components (CC), and the top 5 molecular functions are shown. **(D)** The top 10 KEGG pathways of DEGs.

To investigate the expression features of cirrhotic tissues, the FindMarkers function with default parameters (logfc threshold = 0.25) was applied to identify DEGs in GSE136103 between the two groups. A total of 191 DEGs were found. The heatmap and the volcano plot of DEGs were shown in **Figures 3A, B**. We further performed GO and KEGG analyses of the DEGs (**Figures 3C, D**). These terms were mainly related to immune cell activation, T cell differentiation, NK-cell mediated cytotoxicity, and antigen processing and presentation. Among the DEGs, the expression levels of some IRGs, such as FYN, IFNG, KLRD1, and HLA-G which are related to the process of NK-cell mediated cytotoxicity; some transcription factors (TFs) such as ID2, ETS1, IRF1, and PRDM, which are essential for the development and differentiation of NK cells were decreased in the cirrhotic group. The change in these IRGs and TFs may contribute to the decrease in the NK-cell population (**Supplementary Figures 1**, **2**)

### IRGs of Liver Cirrhosis

**Figure 4:**
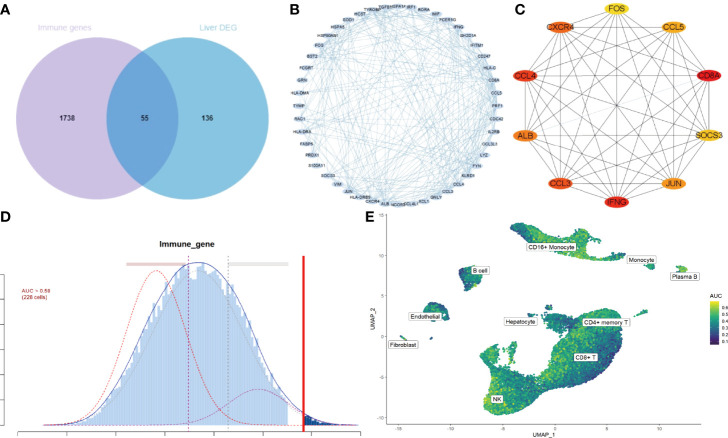
IRGs and IRG scores of cirrhosis from scRNA sequencing data. **(A)** Venn plot showing IRGs of DEGs from the GSE136103 dataset and the gene set of the ImmPort database. A total of 55 IRGs were found. **(B)** The PPI network of the IRGs. **(C)** Results of the CytoHubba plugin and expanded the subnetwork. The color change from yellow to red was indicative of the rank of protein, where deeper red staining indicates higher protein rank. **(D)** Score of 55 IRGs. The threshold was chosen as 0.58. **(E)** UMAP plots of the IRG score in all clusters. CD16+ monocytes and NK cells express more genes and exhibit higher AUC values.

To investigate the IRGs expression characteristics in cirrhotic patients, DEGs were screened to generate IRGs based on the ImmPort database, which summarizes IRGs from published studies. The number of overlapping IRGs between the ImmPort database and DEGs was 55 (**Figure 4A**). The PPI network was constructed to show the relationship between IRGs (**Figure 4B**). The top ten hub IRGs including CD8A, IFNG, CCL4, CCL3, CXCR4, ALB, JUN, CCL5, SOCS3, and FOS were selected. These genes may play critical roles in the process of liver fibrosis (**Figure 4C** and **Supplementary Table 1**). To investigate the IRGs expression characteristics, the IRGs activity of each cell line was identified using the AUCell R package (**Figure 4D**). Cells expressing more genes exhibited higher AUC values, and these cells were mainly in CD16+ monocytes and NK cells (**Figure 4E**).

### DEGs of Liver Cirrhosis From RNA Microarray Expression Data

**Figure 5:**
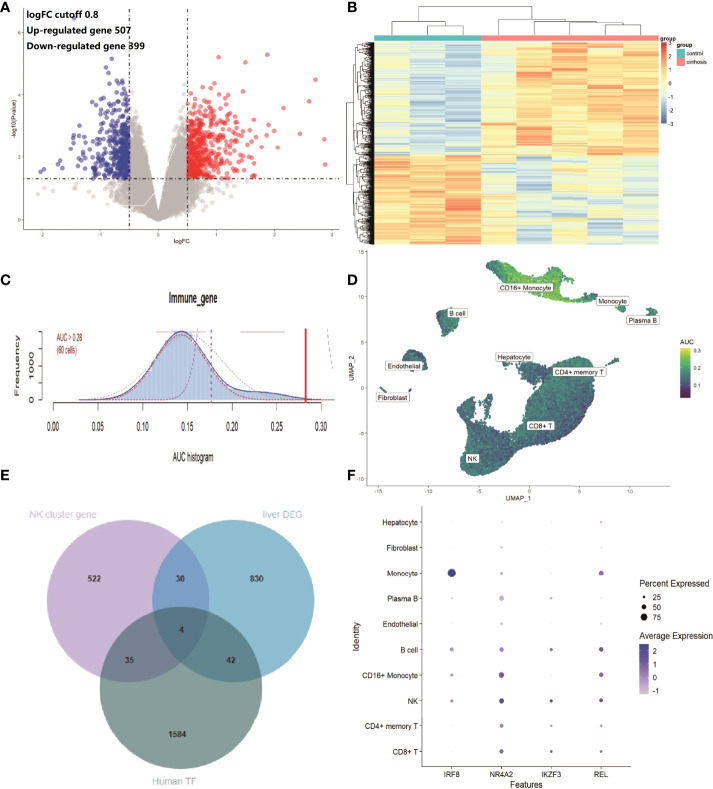
IRGs and relevant regulatory TFs of cirrhosis from the GSE45050 dataset. **(A)** Volcano plot of DEGs (|logFC| > 0.8 and adjusted P value < 0.05). Up-regulated genes are colored red and down-regulated genes are colored blue. **(B)** Heatmap of the top 100 up-regulated and top 100 down-regulated DEGs. **(C)** Score of 103 IRGs. The threshold was chosen as 0.28. **(D)** UMAP plots of the IRG score in all clusters. CD16+ monocytes and NK cells express more genes and exhibit higher AUC values. **(E)** Venn plot showing TFs in the NK cluster of the GSE136103 dataset, Human TF database, and TFs in DEGs of the GES45050 dataset. **(F)** Dot plot of the 4 identified common TFs.

To confirm the expression features of liver tissues in cirrhosis, the RNA microarray expression dataset GSE45050, which included 5 cirrhotic patients and 3 controls, was analyzed to explore DEGs in liver cirrhosis and screen the IRGs. A total of 507 up-regulated and 399 down-regulated DEGs were retained (**Figure 5A** and **Supplementary Table 2**). A heatmap of the top100 up-regulated and top100 down-regulated DEGs is shown (**Figure 5B**). There were 103 overlapping IRGs between the ImmPort database and DEGs (**Supplementary Table 3**). The IRGs activity of each cell line was also identified (**Figure 5C**), and the cells that exhibited higher AUC values were also mainly in CD16+ monocytes and NK cells (**Figure 5D**). To investigate the transcriptionally regulated activity of IRGs, a list of 1,665 TFs was obtained from TFDB (http://bioinfo.life.hust.edu.cn/HumanTFDB/#!/).Four common TFs, IRF8, NR4A2, IKZF3, and REL were identified, which were simultaneously the marker genes of the NK cluster and the DEGs of liver tissues. (**Figures 5E, F**).

### ScRNA Profiling of Cirrhosis by Different Causes

**Figure 6:**
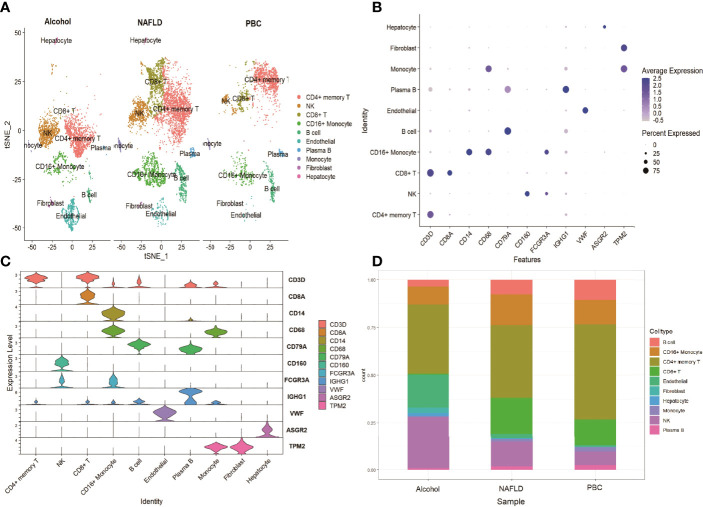
scRNA analysis of cirrhosis by different causes. **(A)** tSNE projection of the alcohol group, the NAFLD group and the control group. **(B)** Dot plot of cell type marker genes. **(C)** Violin plot depicts the distributions of cell type marker genes in each cluster using density curves. **(D)** Cluster distribution in the three groups.

To investigate the expression features of liver leukocytes in different causes of cirrhosis, the scRNA sequencing dataset GSE136103 of 5 cirrhotic samples, including 11,974 cells was further analyzed. Of these five cirrhotic samples, two samples, including 6089 cells, were caused by non-alcoholic fatty liver disease (NAFLD), two samples, including 3576 cells, were caused by alcohol, and one sample, including 2309 cells, was caused by primary biliary cholangitis (PBC). After identifying the cell lineages of every cluster according to marker genes, ten clusters were visualized using the t-SNE algorithm. The CD8+ T cells were decreased in all three groups, and significantly decreased in the alcohol group (0.53%) and the PBC group (13.7%). The reduction in the NK cells cluster was most prominent in the PBC group (7.1%), followed by the NAFLD group (13.1%). The CD4+ memory T cells cluster exhibited a prominent increase in all three groups (49.9% of the PBC group, 37.9% of the NAFLD group, and 36.3% of the alcohol group (**Figure 6A**). The expression of cell type marker genes is shown in the dot plot (**Figure 6B**) and Violin plot (**Figure 6C**). The cell proportions of each cluster in the three groups are shown in **Figure 6D**.

## Discussion

Liver fibrosis is preceded by inflammation. Immune systems play a vital role in regulating the fibrogenic process. Hepatocyte necrosis and HSC activation are major initiators. Activated HSCs secrete TGF-β, which is a crucial pro-inflammatory and pro-fibrogenic factor. The TGF-β/Smad signaling pathway is the classical fibrogenic pathway. Tissue macrophages are attracted by the CCL2-CCR2 axis and phagocytose necrotic hepatocytes and decrease ECM degradation by regulating the expression of tissue inhibitors of metalloproteinase (TIMP). Moreover, TLR4 signaling promotes fibrogenesis by activating HSC, secreting adhesion molecules to recruit macrophages, and boosting TGF-β signaling. The crosstalk between persistent liver injury and the immune response, and the interactions between liver cells and immune cells perpetuate fibrogenesis.

The T cell immune response is closely associated with liver inflammation and viral clearance after hepatitis virus infection. However, evidence indicates that T-cell immunity can also influence the fibrosis process (12). Previous studies reported that transferred CD8+ T cells contributed to liver fibrosis, and CD8+ T cells were found to be able to mediate the direct activation of HSCs in murine models (13). Another study reported that hepatic fibrosis leads to the accumulation of liver resident IL10+ cells, and that these cells could directly impair CD8+ T cell functions and result in the development of hepatocellular carcinoma. CD4+ T cells activity mediates the progression of liver fibrosis by intrinsic apoptosis (14), by secreting signature cytokines IL-4, IL-10, and IFN-γ (15), and by stimulating other immune cells such as NK cells (16). Muhanna et al. analyzed T cells distribution in cirrhotic tissues from 25 HCV patients, seven HBV patients, and six healthy controls (4). The study found that CD4+ T cells and the CD4/CD8 ratio were decreased in cirrhotic tissue, while the difference in intrahepatic CD8+ T cells between the two groups was not significant. In this study, the proportion of CD8+ T cells decreased in the cirrhotic group; however, an increased proportion of CD4+ T cells, including CD4+ effector T cells and CD4+ memory T cells, was found in the cirrhotic group. This result is contrary to that of the study of Muhanna, and there could be several reasons. First, in the study of Muhanna, cirrhotic tissues were obtained by liver biopsy, which could not represent immune microenvironment changes in the entire liver. In this study (7), cirrhotic tissues were obtained from patients who underwent liver transplantation. The tissues were relatively complete and could reflect the complete landscape of immune cell changes in cirrhotic tissues. Second, the etiologies of cirrhosis in the two studies were different. In this study, the causes of cirrhosis were NAFLD, alcohol, and PBC, while in the study of Muhanna, the cirrhotic tissues came from patients with HBV or HCV infection. The immune mechanisms of the fibrosis process caused by different etiologies are not the same. Changes in T cell populations are likely to be dependent on the underlying etiology that drives the fibrosis process.

NK cells are a subgroup of cytotoxic cells of the innate immune system and participate in regulating various liver diseases (17). NK cells with activating receptors such as NKG2D, can be activated to initiate apoptosis of other cells, and release inflammatory cytokines such as IFN-γ, to stimulate other immune cells (18). Numerous studies have indicated that NK cells manifest an anti-fibrotic effect by exerting cytotoxicity to activated HSCs (6, 19). In addition, IFN-γ secreted by NK cells is another vital factor contributing to the anti-fibrotic effects of NK cells. IFN-γ not only inhibits HSC activation and ECM synthesis directly (20) but also amplifies NK-cell cytotoxicity against HSCs by promoting NKG2D expression on liver NK cells to attenuate liver fibrosis (21). A decreased frequency of NK cells with a reduction of function can be observed in the liver of both murine cirrhotic models (22) and cirrhotic patients (4, 23, 24). In this study, the proportion of the NK cluster cells decreased significantly in the cirrhotic group, which was consistent with previous findings. Thus, targeting NK cells may shed light on the treatment of liver fibrosis.

Enrichment analysis of DEGs between the cirrhotic group and the control group mainly focused on the regulation of immune cell activation and differentiation, NK-cell mediated cytotoxicity, and antigen processing and presentation, and these immune reaction pathways may be associated with the fibrosis process. We further investigated the IRGs of DEGs and the top 10 hub genes of the PPI network. Among these hub genes, some are cytokines and chemokines closely related to liver fibrosis (IFNG, CCL3, CCL4, CCL5, and CXCR4) (25, 26). JUN and FOS are transcription factors and the members of the MAPK signaling pathway. They are involved in TGF-β/Samd pathway transduction (27) and can positively regulate HSC proliferation and the progression of fibrosis (28). SOCS3 is a member of the suppressor of cytokine signaling family and has a negative regulatory effect on cytokines such as IFN-γ (29). IRGs are essential for immune reactions and immune infiltration. The variation in these genes also reflected the changes in the immune microenvironment of liver fibrosis.

The IRGs were enriched in the pathway of NK-cell mediated cytotoxicity, suggesting a potential role of NK cells in cirrhosis. In addition, the IRG scores were calculated according to the expression of IRGs, and high scores were mainly found for CD16+ monocytes and NK cells in both scRNA data and RNA microarray expression data. We further explored the potential regulatory mechanisms by investigating TF DEGs in the gene set of the NK cluster. A total of 4 common TFs were found in both the NK cluster and DEGs of liver RNA microarray expression data. IRF8 is a transcription factor of the IFN regulatory factor family that regulates the expression of IFN. IKZF3 is a member of the zinc family, and its encoding protein is an important TF involved in the regulation of lymphocyte development. Studies have shown that the loci of IKZF3 is associated with PBC (30). The encoding protein of REL is the subunit of NF-κB, and the NF-κB signaling pathway has particular relevance to liver fibrosis (31). NR4A2 is a member of the orphan nuclear receptor family, and the overexpression of NR4A2 suppresses the activation of HSCs and ECM production (32). NK cell immune reaction and these genes may play critical roles in the process of liver fibrosis

As previously mentioned, the immune mechanisms of fibrosis caused by different etiologies are not the same; therefore, we further explored the immune cell changes in cirrhotic patients caused by NAFLD, alcohol, and PBC. NAFLD is hallmarked by hepatic steatosis and is tightly associated with inflammation and insulin resistance. NK-cell activities attenuate fibrosis progression of NAFLD by regulating cytokine production (33, 34) and the immune response of other immune cells (35). Our study showed NK cells were decreased in the NAFLD group, and targeting NK cells may be a feasible therapeutic strategy for NAFLD. Excessive alcohol consumption affects cellular immunity. Early studies already indicated that alcohol abuse resulted in reduced T cell numbers (36, 37). Alcohol exposure disrupted the balance between different T cell subsets leading to a decreased frequency of naïve CD4+ T cells and CD8+ T cells, as well as an increased frequency of memory T cells (38, 39), and this conclusion was further supported by our results. It is striking that CD8+ T cells were significantly decreased in the PBC group. Generally, it is thought that CD8+ T cells activation and infiltration are mediators of bile duct damage, and reports have demonstrated that special differentiated CD8+ T cells are increased in PBC patients (40, 41). Further studies are urgently needed to explore the changes in the overall level of T cell subsets and detailed immunologic mechanisms.

In the present study, the scRNA sequencing data GSE136103 was used, which came from the study conducted by Ramachandran et al. (7). The study isolated all hepatic non-parenchymal cells (NPCs) and analyzed the microenvironment of human liver cirrhosis to provide a spatial map and a conceptual framework of liver fibrosis. Our study only analyzed and devoted attention to immune cells (CD45+ NPCs) and divided these cells into more detailed immune subpopulations to explore the immune microenvironment change in cirrhosis, which was a supplement to the original research. However, the study had several limitations. First, the scRNA data showed the changes in the numbers of immune cells, but could not reflect their functional changes. Second, the sample size, especially the number of cirrhotic samples of different etiologies was not large enough to draw accurate conclusions.

In conclusion, we proposed that the reduction in the CD8+ T cluster and NK cells, as well as the infiltration of CD4+ memory T cells, contributed to immune microenvironment changes in cirrhosis. The identified DEGs, IRGs, and pathways may play critical roles in the development and progression of liver fibrosis.

## Data Availability Statement

The datasets presented in this study can be found in online repositories. The names of the repository/repositories and accession number(s) can be found in the article/**Supplementary Material**.

## Author Contributions

YL analyzed the study data, and wrote the manuscript. YL and YD performed the bioinformatics analyses and analyzed the data. XWu assisted with data collection and the analysis. XWang and JN conceptualized the study, supervised the research, and edited the manuscript. All authors contributed to the article and approved the submitted version.

## Funding

The work was sponsored by the National Natural Science Foundation of China (grants NO. 81970519).

## Conflict of Interest

The authors declare that the research was conducted in the absence of any commercial or financial relationships that could be construed as a potential conflict of interest.

## Publisher’s Note

All claims expressed in this article are solely those of the authors and do not necessarily represent those of their affiliated organizations, or those of the publisher, the editors and the reviewers. Any product that may be evaluated in this article, or claim that may be made by its manufacturer, is not guaranteed or endorsed by the publisher.

## Supplementary Material

Supplementary Figure 1 Gene expression levels of IRGs (FYN, IFNG, KLRD1, and HLA-G) on t-SNE plots between the healthy group and the cirrhotic group. Red coloration indicates the expression of the genes.

Supplementary Figure 2 Gene expression levels of TFs (ID2, ETS1, IRF1, and PRDM1) on t-SNE plots between the healthy group and the cirrhotic group. Red coloration indicates the expression of the genes.

Supplementary Table 1 IRGs of liver cirrhosis from GSE136103 dataset.

Supplementary Table 2 DEG of liver cirrhosis from GSE45050 dataset.

Supplementary Table 3 IRGs of liver cirrhosis from GSE45050 dataset.
