# Supplementary figures and images for: Identification of Immune Microenvironment Changes and the Expression of Immune-Related Genes in Liver Cirrhosis
Source: Front Immunol. 2022 Jul 12;13:918445. doi: 10.3389/fimmu.2022.918445 (PMC9315064; doi:10.3389/fimmu.2022.918445)

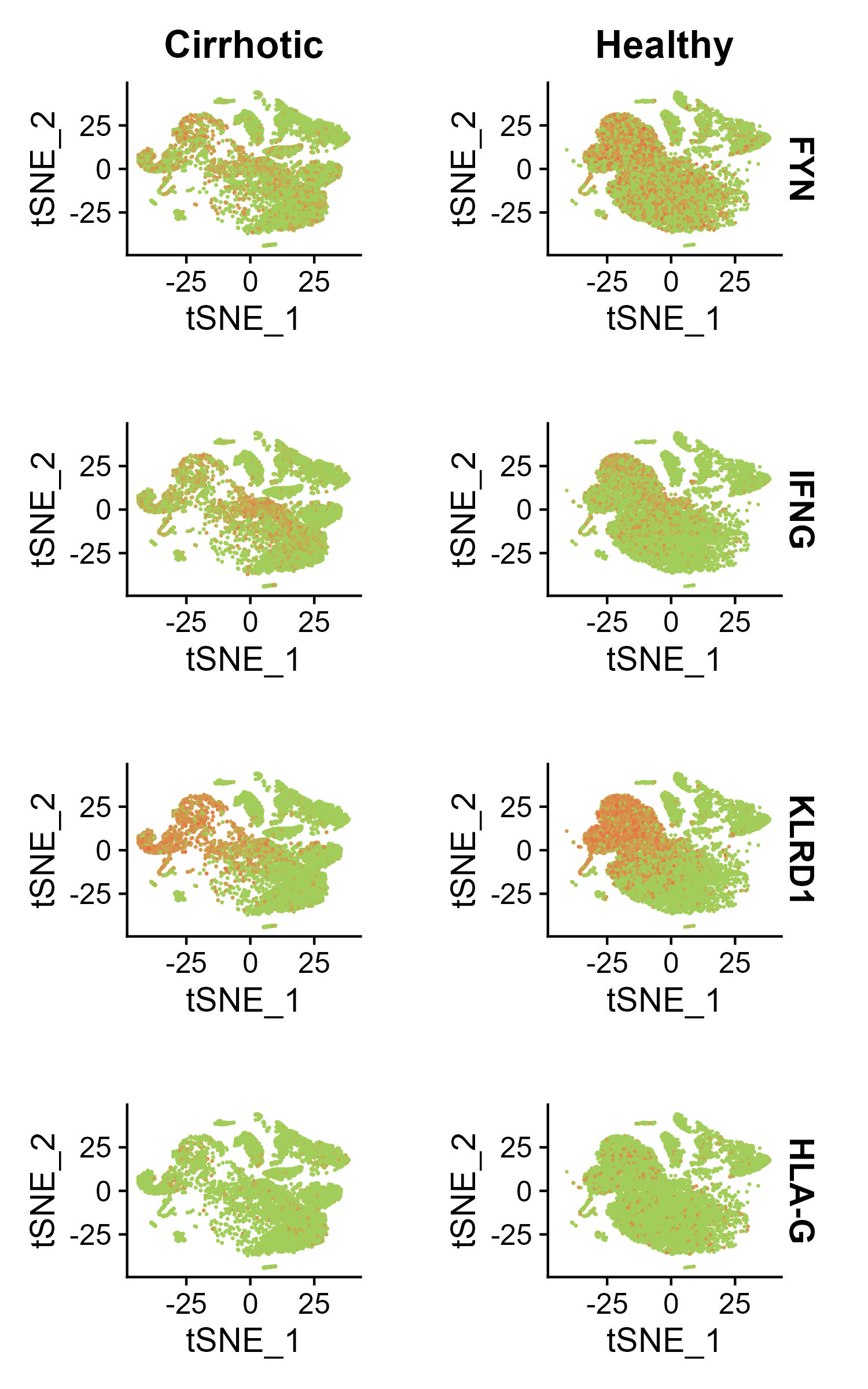

Supplement: Supplementary Figure 1 — Gene expression levels of IRGs (FYN, IFNG, KLRD1, and HLA-G) on t-SNE plots between the healthy group and the cirrhotic group. Red coloration indicates the expression of the genes. [file Image_1.jpeg]

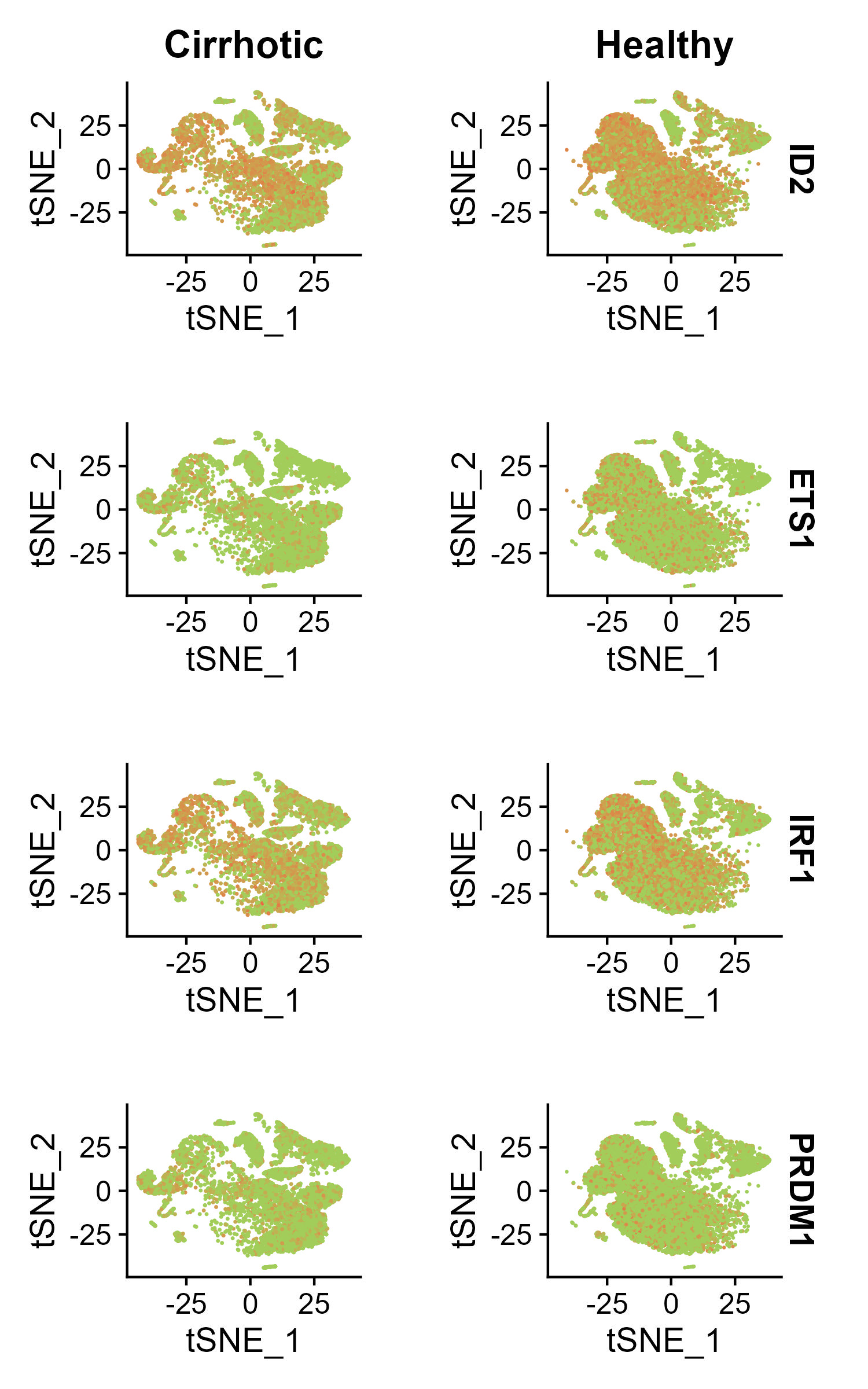

Supplement: Supplementary Figure 2 — Gene expression levels of TFs (ID2, ETS1, IRF1, and PRDM1) on t-SNE plots between the healthy group and the cirrhotic group. Red coloration indicates the expression of the genes. [file Image_2.jpeg]
